# Highly expressed miR-144-3p promotes the proliferation, migration and invasion of colon carcinoma cells by activating the Wnt/β-catenin signaling pathway through targeting SFRP1

**DOI:** 10.7150/jca.87792

**Published:** 2023-09-25

**Authors:** Chunhui Lai, Ningyu He, Jianghui Zeng, Cuizhen Long, Mingfang Shi, Junguo Li, Shengjun Ma, Yu Xiong, Xiuyun Liang

**Affiliations:** 1 Department of Medical Laboratory, The Third Affiliated Hospital of Guangxi Medical University / The Second Nanning People's Hospital, Nanning, Guangxi, China.; 2 Guangxi Key Laboratory of Molecular Immunology Research, Nanning, Guangxi, China.; 3 Department of administrative office, Nanning maternity and Child Health Hospital/Nanning women and children's hospital, Nanning, Guangxi, China.; 4 Department of neurology, The Third Affiliated Hospital of Guangxi Medical University/The Second Nanning People's Hospital, Nanning, Guangxi, China.

## Abstract

**Background:** To investigate the influence of miR-144-3p on the proliferation, migration and invasion of colon carcinoma by targeting secreted frizzled-related protein 1 (SFRP1) as well as of the Wnt/β-catenin signaling pathway.

**Methods:** Based on the TCGA database, the association between the expression of miR-144-3p and the clinical information and prognosis of patients with colon carcinoma was examined, and SFRP1 was selected as the target gene for further studies based on bioinformatics prediction tools. CCK8 assay, wound healing assay and transwell invasion assay were employed to examine the impact of miR-144-3p on colon carcinoma cells. The regulation of SFRP1 by miR-144-3p was investigated using a dual-luciferase reporter system, and a rescue experiment was conducted to further elucidate whether miR-144-3p promotes the migration of colon carcinoma cells through targeting SFRP1 or not. The Wnt/β-catenin signaling pathway-mediated effect of miR-144-3p in colon carcinoma was finally validated through the targeting of SFRP1.

**Results:** The bioinformatics analysis showed that the miR-144 expression levels were substantially greater in colon carcinoma tissue than in para-carcinoma tissue and were closely with clinical stage and prognosis. The findings obtained from the trial indicated that miR-144-3p substantially expressed in colon carcinoma tissue sample and the colon carcinoma cells, and the overexpressed miR-144-3p boosted the colon carcinoma cells' proliferation, migration and invasion. The results of dual-luciferase reporter gene assay revealed that miR-144-3p targeted SFRP1, and rescue experiment was carried out and its results indicated that miR-144-3p increased colon carcinoma cells' migration through targeting SFRP1. In addition, the molecular axis of miR-144-3p/SFRP1 may over-activate the Wnt/β-catenin signaling pathway.

**Conclusions:** The present study has identified a novel malignant biological behavior, namely the ability of miR-144-3p to enhance the proliferation, migration and invasion of colon carcinoma cells by targeting SFRP1 and activating the Wnt/β-catenin signaling pathway. Consequently, miR-144-3p emerges as a promising diagnostic and therapeutic target for colon carcinoma.

## Introduction

As one of the most common malignant tumors, colon carcinoma, accounts for over 1,150,000 new cases and 580,000 fatalities per year 1. Colon adenocarcinoma (COAD), the most commonly-observed pathological subtype of colon carcinoma which primarily affects the intestinal mucosa and extends to other organs, accounts for almost 95% of all cases 2. In recent years, with the implementation of enhanced high-risk factor screening and advancements in relevant diagnostic and treatment techniques, there has been a notable decline in the overall incidence of colon carcinoma. However, there is an alarming trend towards an earlier age of onset and a higher prevalence of advanced stage at definitive diagnosis 3. Although colon carcinoma treatment has advanced greatly because to surgery, chemotherapy, radiation, targeted therapy, and other methods, the prognosis of relevant patients is still unsatisfactory for late diagnosis, rapid disease progression, easy metastasis and poor treatment effect, and the five-year survival rate is only 10% 4, 5. Therefore, it is crucial to understand the molecular mechanism for the emergence and progression of colon carcinoma, and identify the targets and clinical diagnostic signs. The number of studies demonstrating the essential role of MicroRNA (miRNA) in the initiation and progression of malignant tumors has been increasing 6. The binding of miRNA to the 3'-UTR region of target mRNA enables precise regulation of gene expression post-transcriptionally, either by inhibiting translation or inducing mRNA degradation. This pivotal mechanism plays a crucial role in both carcinogenesis and tumor suppression 7. The change of miRNA expression profile in colon carcinoma tissue suggests their potential as diagnostic or prognostic biomarkers 8. For example, the study of Yu et al 9 has indicated that the highly expression of miR-21-5p promotes the proliferation and invasion of colon carcinoma cells by targeting CHL1.The study results of Pan et al 10 have shown that miR-1245a promotes the proliferation and invasion of colon carcinoma by targeting BRCA2. Among numerous miRNAs, miR-144-3p may function as an oncogenic gene, e.g., for thyroid cancer, kidney clear cell cancer, nasopharyngeal carcinoma and etc. 11-13. In addition, miR-144-3p may act as a tumor suppressor gene, e.g., for hepatocellular carcinoma, pancreatic cancer, and etc. 14, 15. The research on the utilization of miRNA-targeted signaling pathways for cancer diagnosis or treatment shows great promise. Although miR-144-3p has been extensively studied in various cancers, there remains a paucity of research regarding its presence and biological function in colon carcinoma, necessitating further exploration of its mechanism of action.

The present study aimed to investigate the involvement of miR-144-3p, SFRP1, and the Wnt/β-catenin signaling pathway in colon carcinoma at multiple levels (i.e., tissue level, cell level, and cellular function) to elucidate their underlying mechanisms of action. Notably, our findings revealed a significant correlation between up-regulated expression of miR-144-3p and both clinical stage and prognosis in patients with colon carcinoma. We demonstrated that SFRP1 was a new target of miR-144-3p, and miR-144-3p promoted a malignant biological behavior of HCT116 cells by targeting SFRP1 and activating the Wnt/β-catenin signaling pathway. The above findings are expected to provide innovative diagnostic and prognostic markers for colon carcinoma, as well as present new opportunities for targeted drug therapy.

## Materials & Methods

### Clinical samples

A total of 60 colon carcinoma patients treated in the Second Nanning People's Hospital from March 2019 to October 2020 were included for colon carcinoma tissue and corresponding para-carcinoma tissue sampling (3 cm or more far from the tumor site). Inclusion criteria included absence of preoperative chemoradiotherapy or immunotherapy, availability of complete clinical and pathological information, and no history or current presence of other malignancies or precancerous lesions. Exclusion criteria comprised patients with severe autoimmune diseases and those with inadequate organ function in vital organs such as the heart, liver, lungs, and kidneys.

The tumor differentiation grade was determined in accordance with the standards of the World Health Organization's tumor classification system. The clinical stages were determined based on the standards set by the American Joint Committee on Cancer. The Second Nanning People's Hospital's Ethics Committee has given its approval for this study (No. Y2018015). The study was conducted in compliance with the ethical guidelines of the Declaration of Helsinki. All participants provided informed consent and this is a retrospective study.

### Cell culture and transfection experiments

Normal colon epithelial cell line NCM460 and colon carcinoma cell line HCT116 were offered by Shanghai Cell Bank, Chinese Academy of Sciences. Both were incubated at 37°C in McCOY's 5A medium (Jiancheng, Nanjing, China) with 5% CO_2_. The Lipofectamine 3000 transfection reagent (Invitrogen, Carlsbad, CA, USA) was used in all of the transfection studies, carefully following the manufacturer's instructions.

### RNA extraction, quantitative real-time polymerase chain reaction(qPCR) and quantitative reverse transcript-PCR (qRT-PCR) detection

The RNeasy FEEP Kit (QIAGEN, Duesseldorf, Germany) was employed to extract total RNA from tissue and cell line according to the operating instructions of the reagent kit. According to the directions for using the reverse transcription kit (Takara, Dalian, China), the extracted total RNA was subjected to RNA reverse transcription. PCR reaction was conducted according to the operating instructions for use of LightCycler 480 SYBR Green I Master kit (Roche, Basel, Switzerland) with tubes in triplicate. Primer information is as follows (all in 5' to 3' directions):

U6 Forward: GGAACGATACAGAGAAGATTAGC; Reverse: TGGAACGCTTCACGAATTTGCG;

miR-144-3p Forward: GGGCGGGTATAGATGATGTACTAA; Reverse: supplied by the Mir-X miRNA qRT-PCR SYBR kit (Clontech);

β-actin Forward: TGGCACCCAGCACAATGAA; Reverse: CTAAGTCATAGTCCGCCTAGAAGCA;

SFRP1 Forward: GGATTGGGCGGAAAGTGAGA; Reverse: TCACACGGAAAGACTGTGGG.

### Immunohistochemistry

The tissue blocks were sliced into 3-4 μm thick sections, followed by dewaxing, antigen retrieval, blocking, and so on. The sections were then incubated overnight at 4℃ with primary antibodies against β-catenin (Abcam, Cambridge, MA, USA, ab223075) at a dilution of 1:2000, SFRP1 (Abcam, Cambridge, MA, USA, ab126613) at a dilution of 1:1000 and Wnt3a (Abcam, Cambridge, MA, USA, ab219412) at a dilution of 1:250. Afterward, a quick enzyme-labeled sheep anti-rabbit IgG polymer secondary antibody (Maixin, Fuzhou, China) was applied, and the samples were observed under a microscope (DM 2500 LED, Leica, Wetzlar, Germany) after color development using DAB (Zhongshan Goldenbridge Biotech Beijing China). Photographic documentation was performed. The evaluation criteria for SFRP1 staining results were based on the presence of brown-yellow granules localized in both the cell membrane and cytoplasm 16. The criteria for β-catenin staining results are based on the method proposed by Maruyama 17 et al: normal staining demonstrates localization to the cell membrane with brown-yellow granules, whereas ectopic expression is observed in the cytoplasm and/or nucleus. The Wnt3a protein-positive reaction was observed to be predominantly localized within the cytoplasm 18. The positive signals were analyzed and quantified utilized the Image J. The statistical results were reported as the ratio of raw integrated density (RawIntDen) to the number of pixels.

### CCK8 assay

According to the manufacturer's instructions for the CCK-8 kit, 24 hours after transfection, each group was transferred to a 96-well plate at a density of 5000 cells per well. Following incubation for 24, 48, and 72 hours, the culture medium in each well was replaced with fresh medium containing 10% CCK-8 (Dojindo, Kumamoto, Japan). After incubating without light for 2 hours, absorbance at a wavelength of 450 nm was measured using a microplate reader (Thermo Fisher Scientific, USA). A cell proliferation curve was produced after statistical analysis of the absorbance measurements.

### Wound healing assay

Inoculate the cells uniformly in a 12-well plate 24 hours after transfection and culture for an additional 24 hours. Once the cell confluence reaches 80%-90%, create scratch lines perpendicular to the bottom of the plate using a consistent force, followed by removal of cells from the lines with PBS. Subsequently, culture the cells in a low serum medium containing 2% FBS (GIBICO, Grand Island, NY, USA). Capture microscopic images using a Leica DM500 microscope (Wetzlar, Germany), and then utilize software to measure and calculate the distance migrated by cells within 24 hours.

### Transwell invasion assay

Dilute Matrigel (Corning, NY, USA) according to the operating instructions of the reagent kit, and spread the dilution on the lower layer of the chamber. Perform the invasion assay after gel is formed. Inoculate HCT116 cells into the upper chamber at a density of 1.2×10^5^ per cell and incubate for 24 h in a 5% CO2 incubator maintained at 37℃. Remove the chamber with filter membrane, and stain the cells attached to the membrane with crystal violet. Count the number of cells which passed through the transwell chamber under 100× magnification for each group.

### Dual-luciferase reporter gene assay

According to the theories of miR-144-3p and SFRP1 alkali base sequence complementarity, the luciferase reporter plasmids consist of wild type and mutant 3'-UTRs based on their respective targets. The interaction between miR-144-3p and SFRP1 was assessed by transfection with the luciferase reporter gene. Colon carcinoma cells were seeded in a 96-well plate and cultured for 24 hours until reaching approximately 50% confluence. Co-transfection was then performed, followed by an additional 48 hours of culture. The relative luciferase activity for each group was determined by calculating the luminous intensity of sea cucumber and firefly luciferases.

### Statistical analysis

SPSS 26.0 statistical software (IBM, Armonk, NY, USA) and GraphPad Prism 10.0 software (La Jolla, CA, USA) were applied to statistical analysis. The mean and standard deviation (xs) of all experimental data were used to express them. The mean of the groups was compared using a one-way ANOVA. If *P*<0.05, difference is considered to be statistically significant. Two groups were compared using the t-test, and three or more groups were compared using a one-way ANOVA. The difference was statistically significant if *P*<0.05.

## Results

### Results based on the bioinformatics prediction and the local cohort

**Figure 1:**
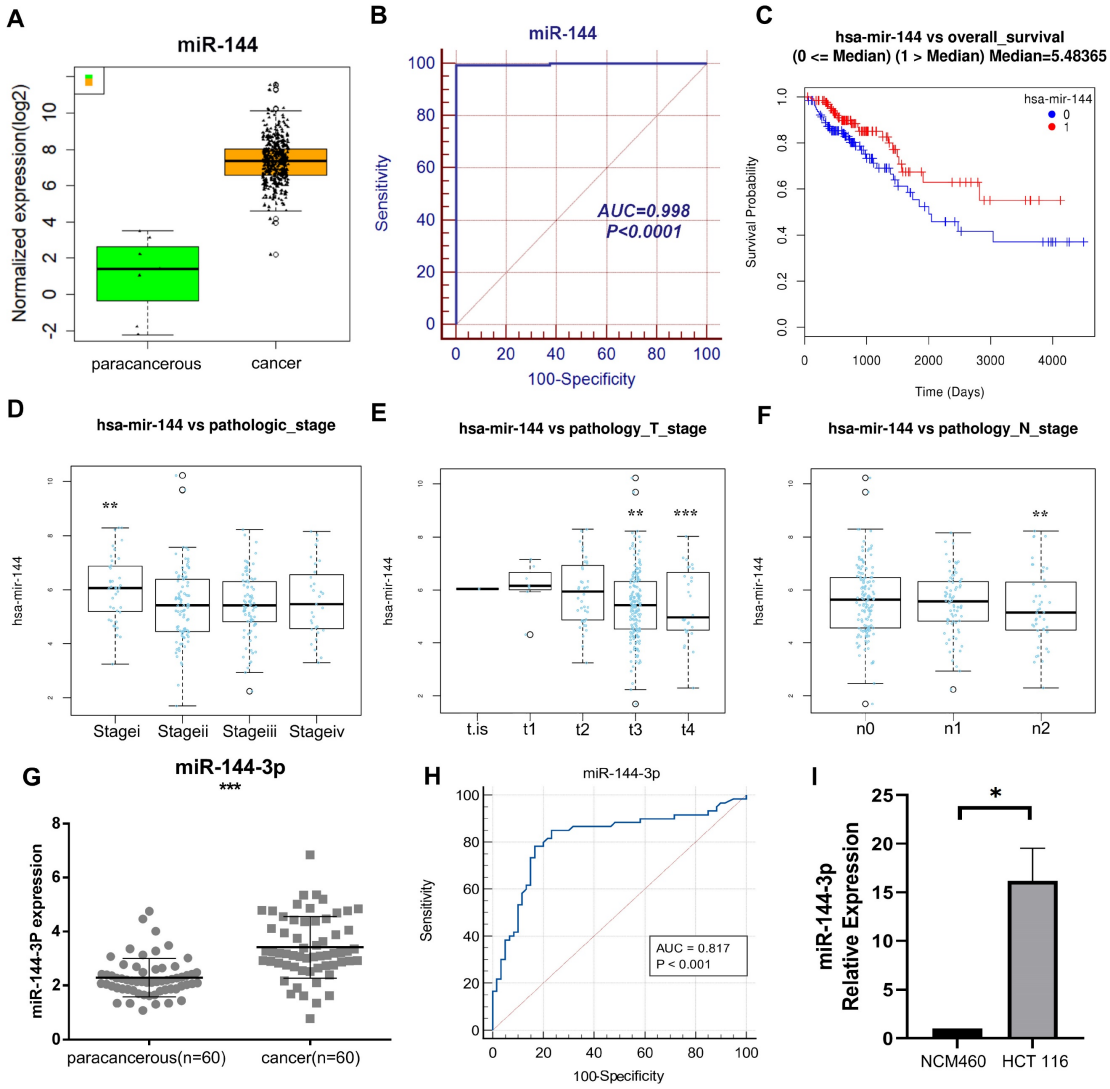
** Clinical significance of miR-144 in COAD based on comprehensive analysis of TCGA data and a local cohort.** (A) Revealing the disparity in miR-144 expression between paracancerous and cancer tissue within the TCGA cohort. (B) ROC curve of miR-144 in TCGA cohort. (C) K-M curve depicting the differential expression of miR-144 in TCGA cohort between high and low levels. (D) Association between miR-144 expression and pathological staging in the TCGA cohort. (E) Association between miR-144 expression and T stage in the TCGA cohort. (F) Association between miR-144 expression and N stage in the TCGA cohort. (G) Differential expression of miR-144-3p in paracancerous and cancer tissues assessed by qPCR in a local cohort. (H) ROC curve of miR-144-3p in local cohort. (I) Revealing the disparity in miR-144-3p expression between NCM460 cells and HCT116 cells. *P<0.05, **P<0.01, ***P<0.001.

Through The Cancer Genome Atlas (TCGA) Genome Data Sharing (GDC) database, our research group obtained mRNA-seq expression data (480 cases of COAD and 41 cases of adjacent normal sample) and miRNA-seq expression data (457 cases of COAD and 8 cases of adjacent normal sample) in April 2018. The data were obtained through TCGA, a community resource project that provides data for research, since the current work followed with TCGA publication standards and data usage policies, clearance from the local ethics committee was not required. A relevant differential analysis showed significant higher expression of miR-144 in cancer tissue than in paracancerous tissue (Fig. 1A), and the receiver operating characteristic (ROC) curve's area under it was 0.998 (Fig. 1B). According to the Kaplan-Meier survival curve, individuals with high miR-144 expression had shorter survival times than those with low miR-144 expression (*P*<0.05, Fig. 1C). In addition, there were differences between clinical stage of tumors in miR-144 expression. The miR-144 expression of stage I was higher than of other stages (Fig. 1D), the miR-144 expression of stages T3 and T4 was lower than of the first three stages (Fig. 1E), and the miR-144 expression at lymph node metastasis stage n2 was lower than at the first two stages (Fig. 1F). These findings suggest that miR-144 exhibits a robust discriminatory capacity between early and late tumor stages. The expression of miR-144-3p was quantified by qPCR in 60 patients with colon carcinoma and adjacent paracancerous tissue (see Table 1 for clinical information on the patients). The results demonstrated that the expression of miR-144-3p in the cancer group was considerably greater than in the paracancerous group (*P*<0.001, Fig. 1G), the area under the ROC curve was 0.817 (*P*<0.001), and the sensitivity and specificity were 85.00% and 76.67%, respectively (Fig. 1H). The expression levels of miR-144-3p in NCM460 cells and HCT116 cells were further detected. The miR-144-3p expression level in HCT116 cells was increased as compared with that in NCM460 cells (*P*<0.05, Fig. 1I). The above results demonstrated that miR-144-3p exhibited significantly elevated expression levels in colon carcinoma and displayed a close correlation with the tumor stage of the corresponding patients.

### The influence of miR-144-3p on colon carcinoma cells

**Figure 2:**
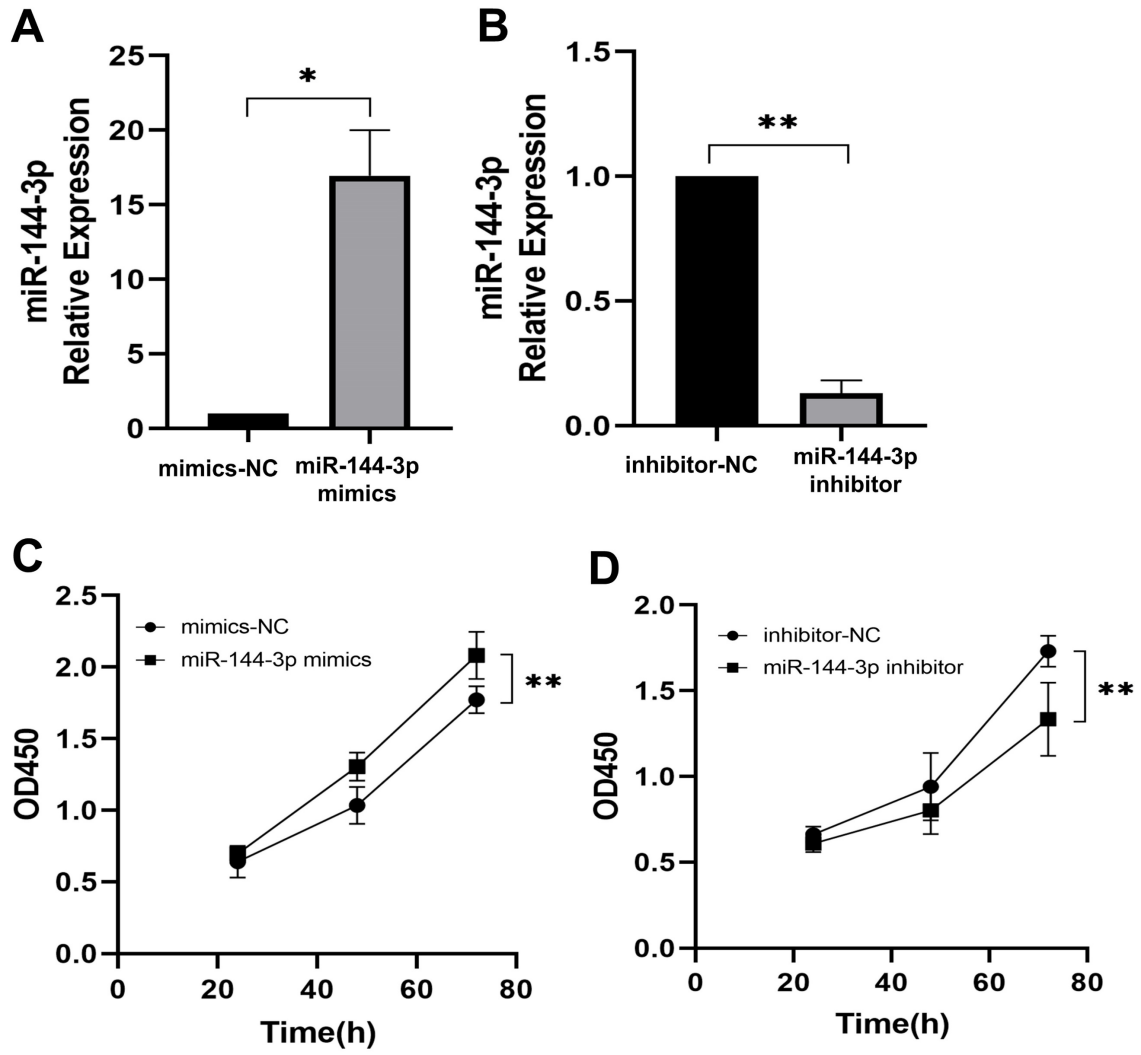
** miR-144-3p promote the ability of proliferation in HCT116 cells.** (A) Expression of miR-144-3p in HCT116 cells transfected with miR-144-3p mimics. (B) Expression of miR-144-3p in HCT116 cells transfected with miR-144-3p inhibitor. (C) Proliferation rate of cells transfected with miR-144-3p mimics. (D) Proliferation rate of cells transfected with miR-144-3p inhibitor. *P<0.05, **P<0.01.

**Figure 3:**
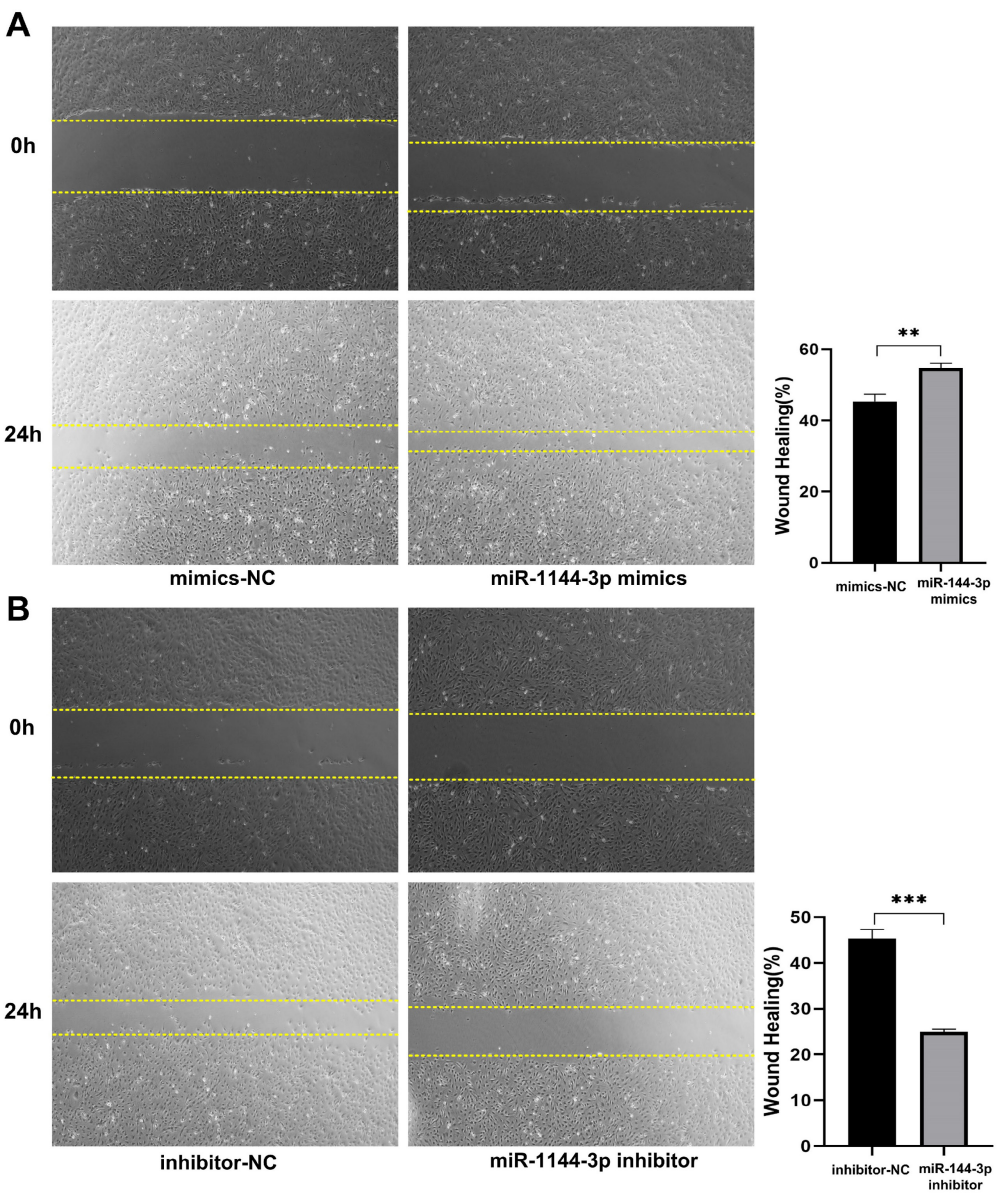
** miR-144-3p promote the ability of migration in HCT116 cells.** (A) Migration ability of cells transfected with miR-144-3p mimics. (B) Migration ability of cells transfected with miR-144-3p inhibitor. **P<0.01, ***P<0.001. Original magnification: 100×.

**Figure 4:**
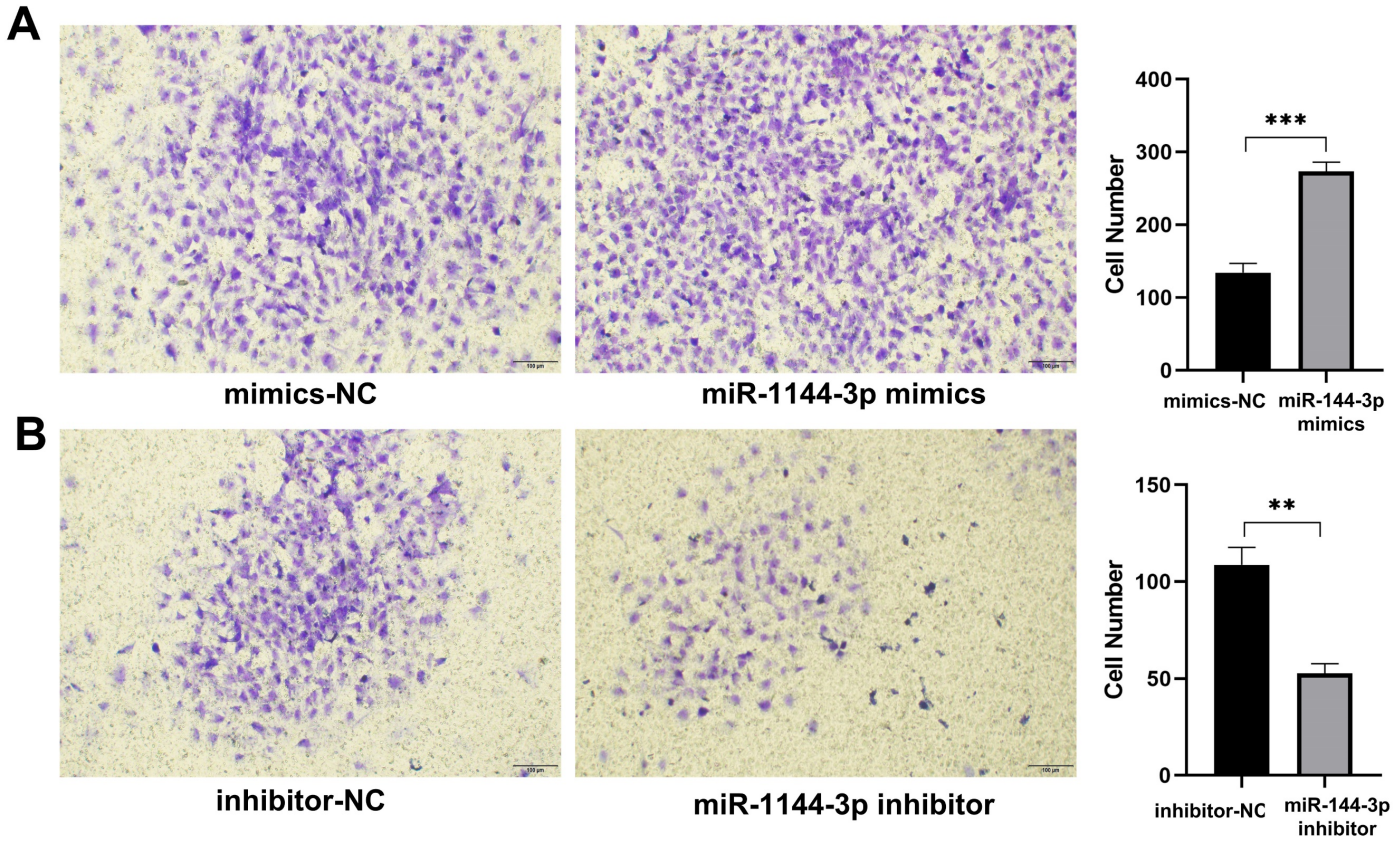
** miR-144-3p promote the ability of invasion in HCT116 cells.** (A) Invasion ability of cells transfected with miR-144-3p mimics. (B) Invasion ability of cells transfected with miR-144-3p inhibitor. **P<0.01, ***P<0.001. Original magnification: 100×.

The qPCR assay was employed to assess the transfection efficiency of miR-144-3p, revealing a significant upregulation in miR-144-3p expression levels following transfection with miR-144-3p mimics in HCT116 cells compared to the negative control (NC) group (*P<*0.05, Fig. 2A). miR-144-3p expression was considerably decreased in contrast to the NC group after miR-144-3p inhibitor transfection into HCT116 cells (*P*<0.01, Fig. 2B). The results of the CCK8 assay revealed a significant enhancement in cell proliferative capacity in the miR-144-3p mimics group compared to the NC group (*P*<0.01, Fig. 2C), and of miR-144-3p inhibitor group was significantly decreased when being compared with the NC group (*P*<0.01, Fig. 2D). After transfection of miR-144-3p mimics in HCT116 cells, the migration ability of the miR-144-3p mimics group was significantly increased at 24 hours compared to the NC group (*P*<0.01, Fig. 3A). Conversely, upon transfection of miR-144-3p inhibitor in HCT116 cells, the migration ability was significantly decreased compared to the NC group (*P*<0.001, Fig. 3B). The results of the transwell invasion assay demonstrated a significantly higher number of transmembrane cells in the miR-144-3p mimics group compared to the NC group (*P*<0.001, Fig. 4A), Conversely, the miR-144-3p inhibitor group exhibited a significantly lower number of transmembrane cells than the NC group (*P*<0.01, Fig. 4B). The aforementioned findings suggest that miR-144-3p may promote the proliferation, migration, and invasion of colon carcinoma cells.

### SFRP1 as the target gene of miR-144-3p

**Figure 5:**
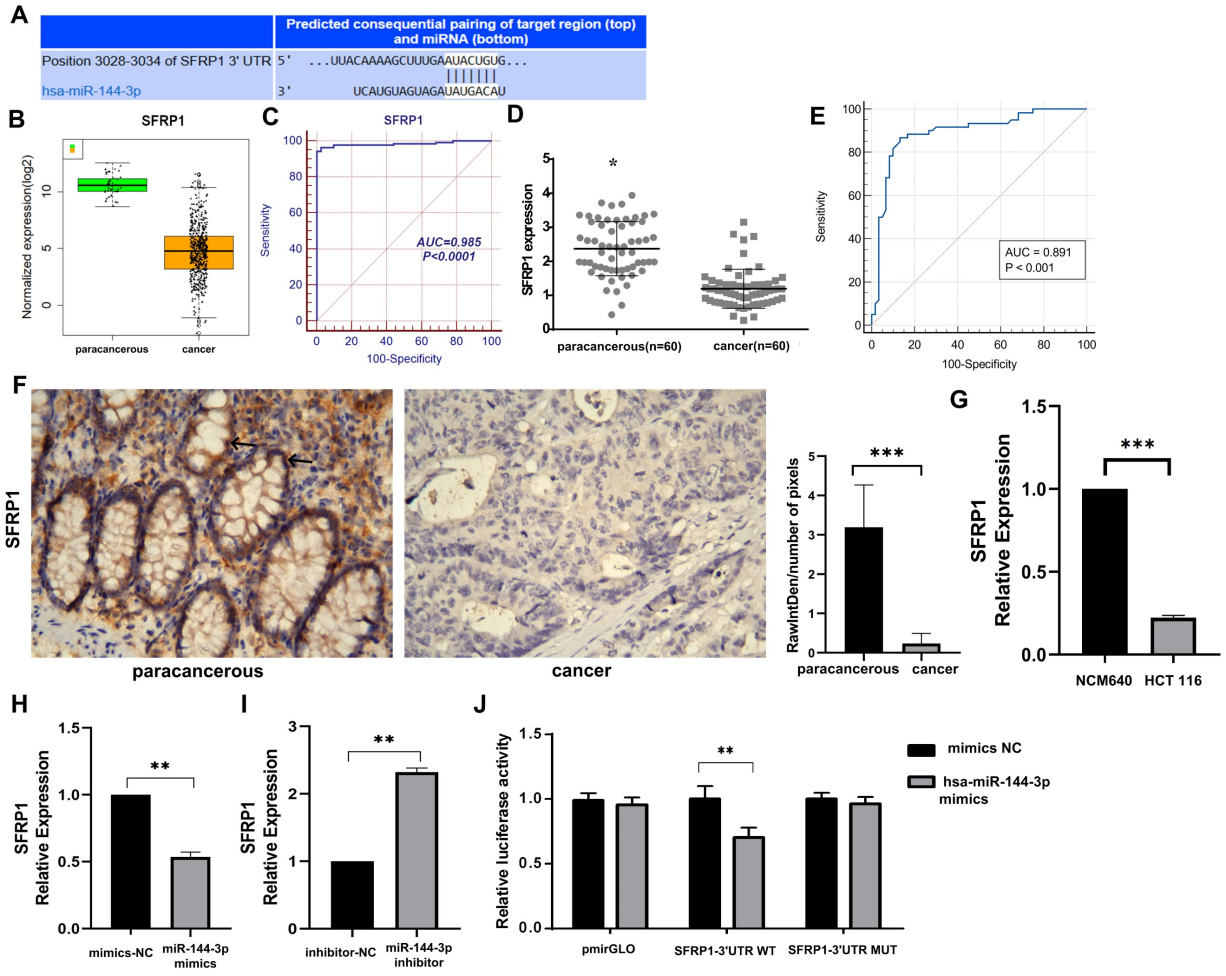
** SFRP1 is a target of miR-144-3p.** (A) The predicted complementary pairing relationship between miR-144-3p and SFRP1 according to TargetScan(the white part represents the binding site of the target region with miRNA). (B) Differential expression of SFRP1 between paracancerous and cancer tissue in the TCGA cohort. (C) ROC curve of SFRP1 in the TCGA cohort. (D) Differential expression of SFRP1 in paracancerous and cancer tissues assessed by qPCR in a local cohort. (E) ROC curve of SFRP1 in local cohort. (F) Immunohistochemistry detect the difference of SFRP1 expression between paracancerous and cancer tissues in local cohort (black arrow showing membrane and cytoplasmic staining). Original magnification: 400×. (G) Revealing the disparity in SFRP1 expression between NCM460 cells and HCT116 cells. (H) The effects of overexpression of miR-144-3p on SFRP1. (I) The effect of knockdown of miR-144-3p on SFRP1. (J) Detection of the targeting relationship of miR-144-3p to the gene SFRP1 with dual-luciferase reporter genes. *P<0.05, **P<0.01, ***P<0.001.

The target gene prediction was performed using the online miRWalk 2.0 software, which integrates twelve databases (miRWalk, Microt4, miRanda, mirbridge, miRDB, miRMap, miRNAMap, Pictar2, PITA, RNA22, RNAhybrid and TargetScan) for accurate identification of potential target genes regulated by miRNAs, and 10 databases predicted that SFRP1 was the target gene of miR-144-3p. The gene was subsequently chosen for further investigation due to the identification of putative miR-144-3p binding sites in the 3'UTR of SFRP1 mRNA through TargetScan analysis (Fig. 5A). The results provided evidence for the predicted complementary pairing between the target gene region of SFRP1 (top) and miR-144-3p (bottom). Furthermore, an analysis on the data concerning expression of TCGA COAD mRNA-seq showed that the expression of SFRP1 in cancer tissue was significantly lower than in paracancerous tissue (Fig. 5B), and the area under ROC was 0.985 (Fig. 5C). The qPCR were used to detect the expression of SFRP1 in 60 patients of colon carcinoma and paracancerous tissue samples. The results revealed a significant down-regulation of SFRP1 expression at the mRNA level in cancer tissue compared to paracancerous tissue (Fig. 5D). Additionally, the area under the ROC was determined to be 0.891 (*P*<0*.*001), with a sensitivity and specificity of 86.67% (Fig. 5E). The immunohistochemical staining results reveal a significantly elevated expression of SFRP1 in the paracancerous tissue compared to the cancer tissue (Fig. 5F). The expression level of SFRP1 in HCT116 cells was lower than in NCM460 cells (*P*<0.001, Fig. 5G). The miR-144-3p mimics group's expression level of SFRP1 was significantly lower than in the NC group (*P*<0.01, Fig. 5H). In the miR-144-3p inhibitor group, SFRP1 expression was noticeably higher than NC group (*P*<0.01, Fig. 5I). The results obtained from the dual-luciferase reporter gene assay demonstrated a significant attenuation in luciferase activity when co-transfecting the wild-type 3'-UTR gene reporter plasmid with miR-144-3p mimics. Conversely, minimal impact on luciferase activity was observed upon co-transfection of the mutant gene reporter plasmid harboring a miR-144-3p binding site mutation with miR-144-3p mimics (*P*<0.01, Fig. 5J). The aforementioned findings indicate that SFRP1 exhibits decreased expression in both colon carcinoma tissue and cells, serving as one of the downstream target genes directly influenced by miR-144-3p.

### The molecular axis of miR-144-3p/SFRP1 for regulating the migration of colon carcinoma cells

**Figure 6:**
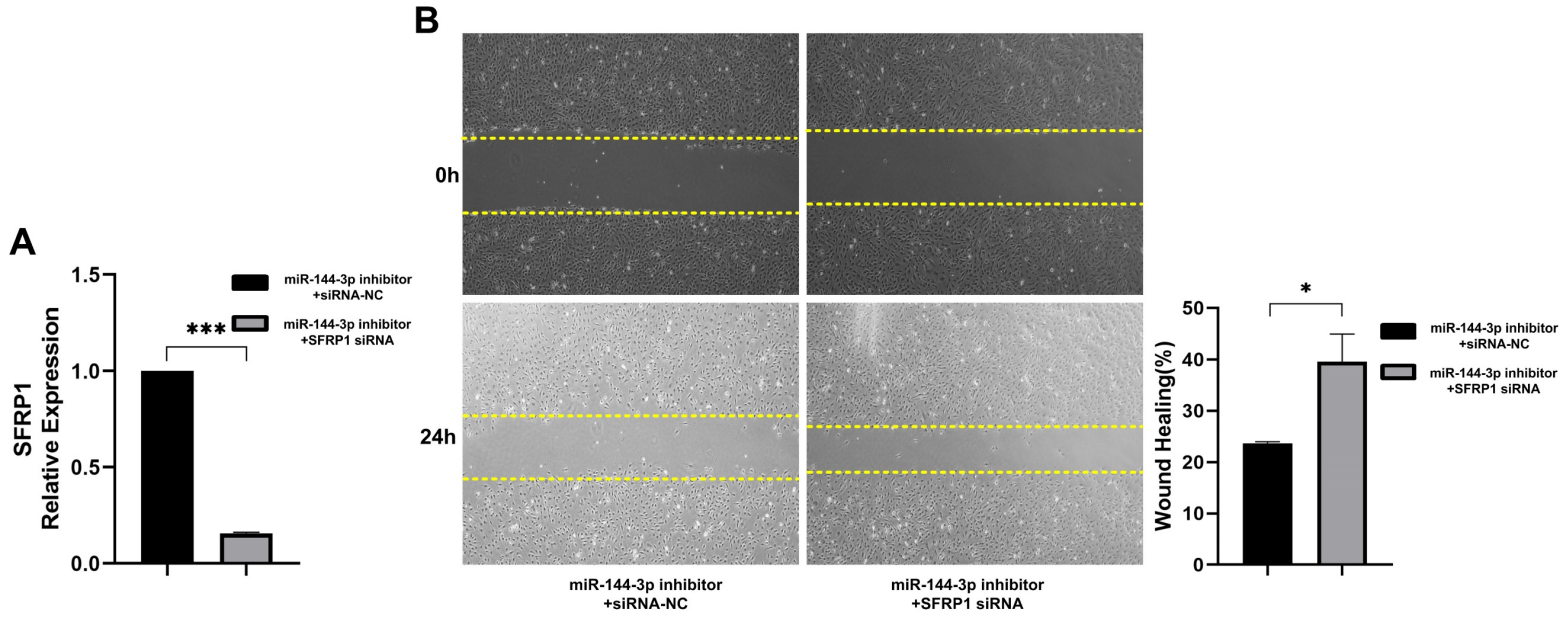
** The miR-144-3p/SFRP1 molecular axis regulates migration of colon carcinoma cells.** (A) Expression of SFRP1 after co-transfection of miR-144-3p inhibitor and SFRP1 siRNA. (B) The migration ability of cells co-transfected with miR-144-3p inhibitor and SFRP1 siRNA was detected by wound healing assay. Original magnification: 100×. *P<0.05, ***P<0.001.

In order to validate the promotion of migration abilities in colon carcinoma cells by miR-144-3p through regulation of SFRP1, a functional rescue experiment was conducted. HCT116 cells were co-transfected with a miR-144-3p inhibitor and SFRP1 siRNA, and the transfection efficiency of the vectors was confirmed using qPCR (*P*<0.001, Fig. 6A). The findings demonstrated that downregulation of SFRP1 counteracted the inhibitory impact exerted by miR-144-3p inhibitor on cellular migratory capacity (*P*<0.05, Fig. 6B). The above results demonstrate that miR-144-3p promotes the migration of colon carcinoma cells by mediating SFRP1.

### The influence of miR-144-3p/SFRP1 molecular axis on Wnt/β-catenin signaling pathway

**Figure 7:**
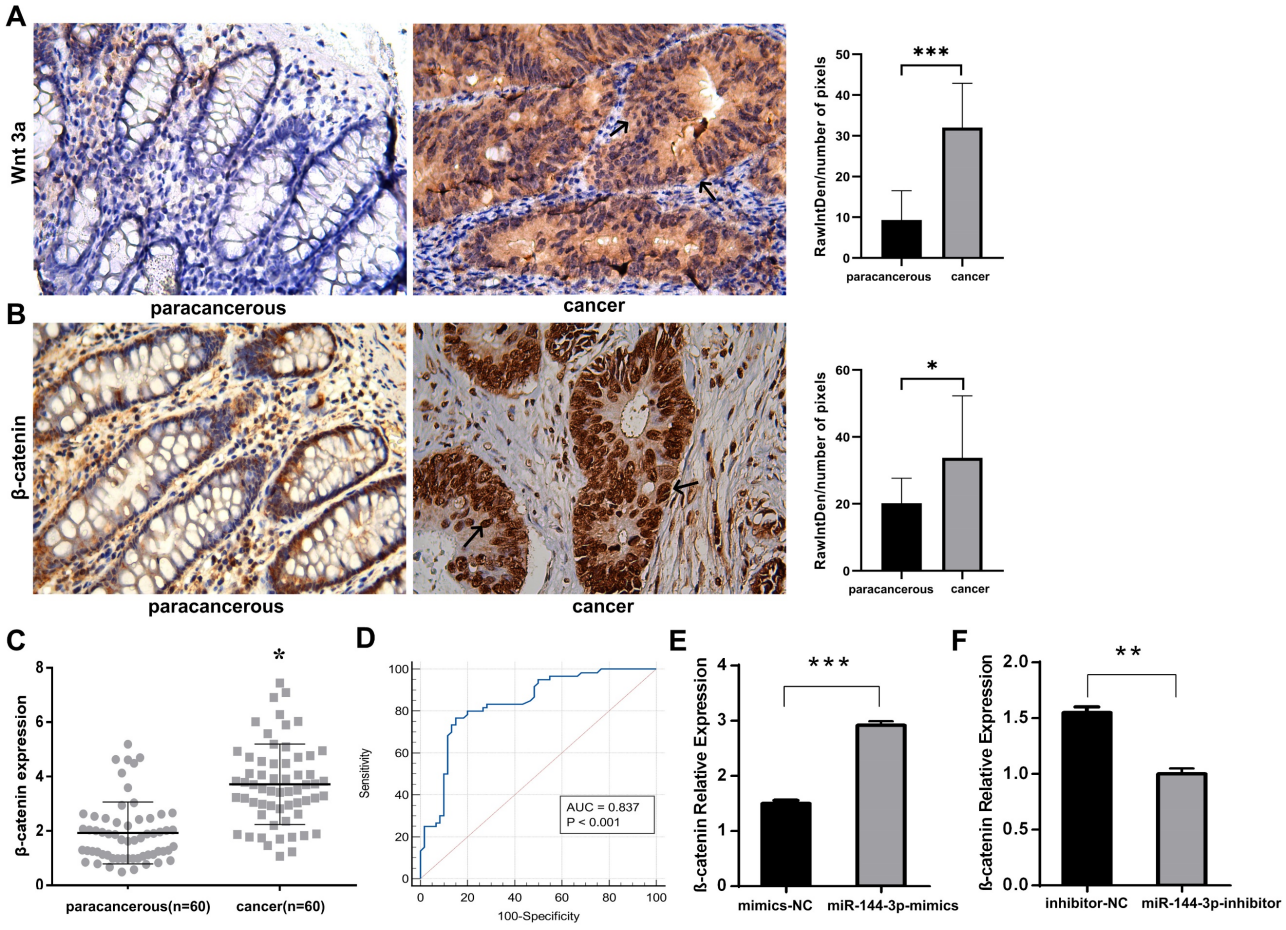
** The miR-144-3p/SFRP1 molecular axis is capable of activating the Wnt/β-catenin signaling pathway.** (A) Immunohistochemistry detect the difference of Wnt3a expression between paracancerous and cancer tissue (black arrow showing cytoplasmic staining). Original magnification: 400×. (B) Immunohistochemistry detect the difference of β-catenin expression between paracancerous and cancer tissue (black arrow showing nuclear staining). Original magnification: 400×. (C) Differential expression of β-catenin in paracancerous and cancer tissues assessed by qPCR in a local cohort. (D) ROC curve of β-catenin in local cohort. (E) The effect of overexpression of miR-144-3p on β-catenin. (F) The effect of knockdown of miR-144-3p on β-catenin. *P<0.05, **P<0.01, ***P<0.001.

The immunohistochemical staining technique was employed to assess the expression of Wnt3a and β-catenin in both cancer and adjacent noncancerous tissues. Our findings revealed elevated levels of the signaling pathway ligand, Wnt3a, as well as the key protein involved in this pathway, β-catenin, within cancer tissue compared to lower expression levels observed in adjacent noncancerous tissue (Fig. 7A,B), indicating an overactivation of the Wnt/ β-catenin signaling pathway. The qPCR was adopted to detect the expression of β-catenin in cancer tissue, and the results indicated that, as compared with the paracancerous group, the expression level of β-catenin in the cancer group was significantly increased (*P<*0*.*05, Fig. 7C). The area under the ROC was 0.837 (*P*<0*.*001), with a sensitivity of 76.67% and a specificity of 85.00% (Fig. 7D). After transfecting HCT116 cells with miR-144-3p mimics or miR-144-3p inhibitor, the expression of β-catenin was quantified using qPCR. The results revealed a significantly higher expression level of β-catenin in the miR-144-3p mimics group compared to the NC group (*P*<0.001, Fig. 7E). Conversely, the miR-144-3p inhibitor group exhibited a significant decrease in β-catenin expression level when compared to the NC group (*P*<0.01, Fig. 7F). The aforementioned findings demonstrated that the potential activation of the Wnt/β-catenin signaling pathway in colon carcinoma tissues and cells through the miR-144-3p/SFRP1 molecular axis.

## Discussion

For colon carcinoma, a disease of intricate pathogenesis that poses a significant threat to human life and health, as well as for the purpose of accurate diagnosis or effective therapy, it is imperative to explore and identify novel biomarkers. With the advent of the era of big data, large public biological databases, e.g., TCGA and Gene Expression Omnibus (GEO), are being improved and updated continuously. More and more researchers are exploring diagnostic or therapeutic biomarkers of colon carcinoma by using bioinformatics methods, and validating them through relevant experiments as well 19, 20. The highly conservative and sequential miRNA expression is complex in tissue specificity, showing different regulatory specificity in types of tumor tissue cells. Some studies have shown that miR-144-3p may promote the proliferation, migration and invasion of tumor cells, thus acting as a tumor promotor. Cao et al 21 have found that miR-144-3p is highly expressed in thyroid cancer tissue and the silencing miR-144-3p can inhibit cell proliferation, invasion and promote cell apoptosis. Also, previous research have proven that miR-144-3p can inhibit biological behaviors like the proliferation, migration and invasion of tumor cells 21, 22. Despite extensive reporting on miR-144-3p in tumors, limited investigations have been conducted regarding its presence in colon carcinoma. In this study, we aim to elucidate the impact and mechanism of miR-144-3p in modulating the Wnt/β-catenin signaling pathway by targeting SFRP1, thereby offering novel biomarkers for the diagnosis, prognosis, and molecular targeted therapy of colon carcinoma. The findings of our study demonstrate the upregulation of miR-144-3p in both colon carcinoma tissue and colon carcinoma cells, consistent with the results obtained from analysis of the TCGA database. Our conclusions are contrary to the reported downregulation of miR-144-3p in colorectal carcinoma (CRC)/colorectal adenocarcinoma (CRA) 23, 24. Both reports have indicated that the down-regulated miR-144-3p may lead to cancer inhibition in CRC/CRA by targeting different target genes. However, an analysis of the the TCGA database found a significant up-regulation of miR-144-3p expression in CRC tissue, while qPCR data showed no significant difference in miR-144-3p expression between CRC and adjacent normal tissue. The miR-144-3p expression in the early stage of CRC was considerably higher than the late stage of CRC, according to only an inter-tumor comparison 8. We speculate that the differences in these studies may be related to factors like colon carcinoma tissue sample and clinical typing, and considering that miR-144-3p acts on different target genes for different effects, the effects of target genes on the signaling pathway vary. Our experimental data have indicated that over-expression of miR-144-3p may significantly promote the proliferation, migration and invasion of HCT116 cells, while the knockdown of miR-144-3p may weaken HCT116 cells' proliferation, migration and invasion. Therefore, we postulated that miR-144-3p may contribute to the progression of colon carcinoma by facilitating disease proliferation.

The Wnt signaling pathways, which regulate cellular processes such as cell division, differentiation, and apoptosis, encompass the canonical Wnt signaling system (Wnt/β-catenin signaling pathway), planar cell polarity Wnt/PCP signaling pathway, and Wnt/Ca^2+^ signaling pathway 25, 26. The Wnt/β-catenin signaling pathway facilitates the binding of Wnt protein to specific receptors of the frizzled (FZD) family, thereby initiating intracellular signal transduction and promoting β-catenin aggregation, ultimately leading to the activation of transcription factor T cell factor/lymphocyte enhancer factor (TCF/LEF) for regulating cellular behavior. The crucial determinant lies in maintaining intracellular β-catenin stability. The activation of the Wnt pathway is contingent upon high levels of β-catenin, while its inhibition occurs when β-catenin levels are low 27, 28. The abnormal regulation and over-activation of Wnt signaling pathways have been demonstrated to be intricately associated with the occurrence and progression of diverse malignancies, exerting a pivotal role in their pathogenesis 29. Some studies on sporadic and hereditary CRC have found that the Wnt signaling pathway is closely associated with the occurrence and development of CRC, and about 90% of CRC cases have this signal transduction pathway regulated abnormally 29, 30. While β-catenin plays a pivotal role in the Wnt/β-catenin signaling pathway, initially characterized for its mutation in colon carcinoma, numerous mutations have been identified across various malignancies 18. Wnt3a is a Wnt protein that stimulates the Wnt/β-catenin signaling pathway 31. Therefore, β-catenin is often used for the research on cancer target therapy. For example, based on proteolytic targeting chimera (PROTAC) technology, Liao et al 32 designed a novel β-catenin reducer, which can sustainably degrade β-catenin with a strong inhibitory effect on Wnt signaling, thus significantly reducing the proliferation of HCT116 cells and inhibiting the tumor formation in nude mice. The immunohistochemistry results of this study indicated that signal ligand Wnt3a and key regulatory factor β-catenin of the Wnt/β-catenin signaling pathway were highly expressed in colon carcinoma tissue cells. The expression of Wnt3a in paracancerous tissue cells was minimal, whereas it exhibited significant cytoplasmic accumulation in colon carcinoma tissue, and β-catenin was only expressed in the cell membrane of the paracancerous tissue, but massively accumulated in the nucleus and cytoplasm of colon carcinoma tissue, indicating that an abnormal over-activation of the Wnt/β-catenin signaling pathway in colon carcinoma cells, which was consistent with the results reported in relevant literature 33. Therefore, we propose that miR-144-3p facilitates the aberrant activation of the Wnt/β-catenin signaling pathway in colon carcinoma.

In the recent past, studies have found that miRNAs are regulated by Wnt signals in various tumorigenesis processes. Interestingly, abnormally activated miRNA in cancer can also automatically regulate Wnt/β-catenin through a positive feedback loop 34. For example, Wnt signaling can induce the expression of miR-146a in CRC, and at the same time, miR-146a can stabilize β-catenin by acting on corresponding target genes, thus forming a chain of rings to maintain the activity of Wnt signaling pathway 35. This regulatory mechanism of interaction has provided a theoretical basis for miRNA and Wnt network factors as biomarkers for disease diagnosis or prognosis and targeted drug therapy 36, 37. This study has shown that in colon carcinoma cells, the over-expression of miR-144-3p significantly increases β-catenin, while knockdown of miR-144-3p significantly reduces β-catenin, suggesting that miR-144-3p can activate the Wnt/β-catenin signaling pathway. The immunohistochemical staining revealed abundant expression of SFRP1 in the cell membrane and cytoplasm of paracancerous tissues, whereas it was not present in cancer tissue. The expression of SFRP1 exhibited an inverse correlation with the levels of β-catenin and Wnt3a in this study. Our findings corroborate the literature's assertion that SFRP1 functions as an extracellular antagonist of the Wnt signaling pathway, competitively inhibiting signal transduction and preventing excessive activation of the Wnt signaling pathway 38. The downregulation of the signal suppressor protein SFRP1 may contribute to the accumulation of β-catenin in the cytoplasm and nucleus of colon carcinoma, as well as the activation of the Wnt/β-catenin signaling pathway. SFRP1 can inhibit the proliferation, migration and invasion of CRC cells and promote cell apoptosis *in vitro* 39. Multiple studies have demonstrated that SFRP1 exhibits preferentially hypermethylated in in colorectal cancer, leading to a significant downregulation of its expression 40, 41. DNA methylation is a biomarker that may be used for diagnosis, prognosis, and therapeutic effect evaluation 42. There have been studies reporting methylation of SFRP1 gene as a diagnostic biomarker in plasma, feces and surgically removed tumor tissues of patients with CRC 43-45.

In this present study, we have demonstrated a significant upregulation of miR-144-3p in colon carcinoma and its correlation with clinical presentation. Besides, miR-144-3p encourages colon carcinoma cell proliferation, migration and invasion through the target gene SFRP1. We have reported a study on the mechanism of miR-144-3p/SFRP1 molecular axis in colon carcinoma through activating the Wnt/β-catenin signaling pathway for the first time. These results have added to the growing body of evidence supporting the molecular axis of miR-144-3p/SFRP1 as a therapeutic target of colon carcinoma. The limitations of our study encompass the absence of protein level verification to evaluate the impact of miR-144-3p on SFRP1, as well as the omission of further investigation into the correlation between immunohistochemical findings and tumor stage. The future research will primarily focus on investigating the intricate relationship between miR-144-3p-mediated targeted regulation of SFRP1, methylation patterns within the promoter region of SFRP1, and subsequent modulation of protein levels. This comprehensive investigation aims to establish a pioneering theoretical framework for advancing colon carcinoma treatment strategies.

## Figures and Tables

**Table 1:** Clinical characteristics of the local cohort

Clinical parameters	Patient cohort (n = 60)
**Sex**	
Female	28
Male	32
**Age**	
Below 60 years	20
Above 60 years	40
**Nation**	
Hanzu	41
Zhuangzu	17
Yaozu	2
**Histological Subtype**	
Adenocarcinoma	42
Tubular adenocarcinoma	15
Mucinous adenocarcinoma	3
**Clinical stage**	
I-II	21
III-IV	39
**Lymph node metastasis**	
Absence	22
Presence	38
**Distant metastasis**	
Absence	41
Presence	19

## Abbreviations

CRC: colorectal carcinoma
CRA: colorectal adenocarcinoma
COAD: Colon adenocarcinoma
GDC: Genome Data Sharing
GEO: Gene Expression Omnibus
LEF: lymphocyte enhancer factor
miRNA: MicroRNA
PROTAC: proteolytic targeting chimera
qPCR: quantitative real-time polymerase chain reaction
qRT-PCR: quantitative reverse transcript-PCR
ROC: receiver operating characteristic
SFRP1: secreted frizzled-related protein 1
TCF: T cell factor
TCGA: The Cancer Genome Atlas
